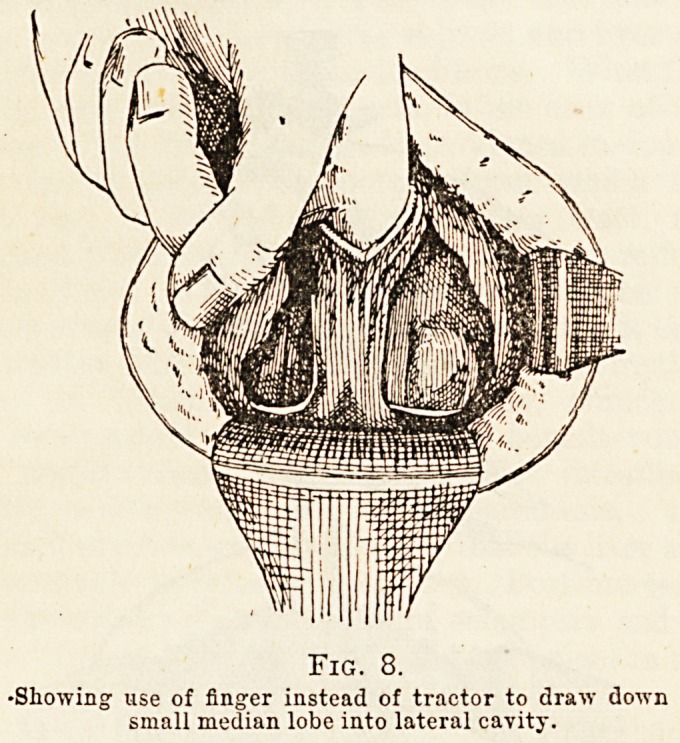# Perineal Prostatectomy

**Published:** 1908-04-18

**Authors:** 


### SPECIAL ARTICLE.

### PERINEAL PROSTATECTOMY.

(*Concluded from page* 36.)

### External and Internal Enucleation.

Everything is now in readiness for the external enucleation—that is to say, the separation of the capsule from the lateral lobes by the blunt dissector. It is important to start the separation in the right layer, not so deep as to lead into the substance of the lobe, and not so superficially as to be outside of

Fig. 6.
**Enucleation of lobes. Forceps in position.**

the most of the capsule. The stripping up process is continued by blunt dissectors.

The internal enucleation—that is to say, the separation of the prostate from the urethra, should be taken up after the external, as it is a much more delicate procedure and often requires considerable care to prevent tearing into the urethra. As remarked above, the primary incision is made with the scalpel until past the level of the urethra, after which the blunt dissector is used. During this procedure the shaft of the prostatic tractor is firmly grasped in the operator's left hand; it serves not only to draw the prostate so well down into the cutaneous wound that every procedure is done in plain view, but also to steady the prostate and to mark out the course of the urethra so that it can be avoided. At the apex of each lateral lobe firm adhesions to the capsule, usually requiring division with scissors, are nearly always present.

When the enucleation of a lateral lobe has progressed fairly well on each side, it is advantageous to have traction made on the lobe itself; for this purpose Mr. Young has devised special fenestrated forceps. The two blades grasp the prostate with broad surfaces, so shaped as to hold, but not to cut the lobe when pressure is applied (Fig. 6).

The lobes usually come out each in one piece, and it is possible to apply considerable traction without tearing them, thus greatly facilitating the deeper enucleation. Much of the enucleation is done with the blunt dissector, but when the intravesical portion of the lateral lobe is reached the finger may often be used so as to avoid tearing through the thin mucous membrane covering it.

The intravesical blade of the prostate tractor, which can be distinctly palpated through the mucous membrane by the enucleating finger, serves to direct the separation of the deeper portion, and warns against tearing into the bladder. It also shows when some of the lobe has been left behind. The condition present after the enucleation of the two lateral lobes leaves the empty capsule on each side, and the bridge of tissue surrounding the ejaculatory ducts and the urethra intact in the centre.

### Removal of Median Lobe.

After the lateral lobes have been shelled out, attention should be directed to the median portion of the prostate. There is often a more or less extensive hypertrophy of the prespermatic group of glands, and the mass can be easily seen, or felt by the finger in one of the intracapsular cavities (Fig. 7).

Further examination will generally reveal a fair amount of tissue between the median lobe and the ejaculatory ducts. The median enlargement is generally attached to one or both of the lateral lobes, so that there is no difficulty in shelling it out

Fig. 7.
**Showing technique of delivery of middle lobe into cavity of left lateral lobe.**

through one of the lateral cavities, without disturbing the integrity of the ejaculatory ducts. (Fig. 8.)

The prostatic tractor may be used with great advantage in removing a median lobe, and the technique for drawing it down into one of the lateral cavities where it can be enucleated is as follows:—Push the tractor backwards until free in the bladder cavity, depress the handle of the instrument so that the shaft can lie on the top of the middle lobe, and then rotate the instrument 90 degrees so that one of the blades projects downward behind it. Outward traction should then engage the lobe, and it can be drawn down so as to come into sight. To get it into one lateral intracapsular cavity, say the left, two manœuvres are of help: Pushing against it with the index finger of the left hand, which has been inserted into the right intracapsular cavity, as seen in Fig. 8, and rotation of the blade engaging the middle lobe in the same direction, traction being made on it all the while. When the median lobe presents in the left intracapsular cavity, the operator turns the tractor over to an assistant who continues the traction, while he grasps the lobe with the forceps described above, and then rapidly enucleates it.

Often the median mass is directly continuous with the left lateral lobe, and can be removed with it.

The condition present after the enucleation of a median lobe shows that the median cavity communicates with the lateral cavities on each side, beneath the intact urethra, whilst the seminal ducts are separated off by the posterior capsule.

Before withdrawing the tractor a careful examina-

Fig. 8.
Showing use of finger instead of tractor to draw down small median lobe into lateral cavity.

tion should be made by inserting the finger into both of the lateral cavities and palpating the blades through the vesical mucosa, in order to determine that no important glandular mass has been left behind. The tractor is then removed by first rotating the blades until they come together and then withdrawing the instrument.

### Provision for Drainage.

Abundant vesical drainage should be provided, as a small tube may easily become plugged by blood clots, and thus prove useless.

Two catheters of fairly good size are fastened together by ligatures before the operation, so that as soon as the tractor is withdrawn they can be inserted through the perineal wound into the urethra and bladder. In order to facilitate their introduction it is best to cut obliquely across the end of each catheter and then fasten the cut surfaces together with a single suture, thus making a common point for the two catheters. One catheter is immediately connected with a tank of normal salt solution at body heat, and the bladder thoroughly washed clean of blood.

After the tubes have been properly adjusted, they are tied by a heavy silk suture to the skin at the upper angle of the wound. The lateral prostatic cavities are then firmly packed each with a small strip of gauze, but care is taken that the packing is confined to the lateral cavities of the prostate and especially that none may be allowed to press against the rectum.

Before closing the cutaneous wound one should always examine the rectum. With a gloved finger inserted through the anus and another in the wound the rectal wall should be carefully examined. Above the anal sphincter it is usually thin, even when uninjured. When it has been very adherent to the prostate the muscular tunic may be torn, and it should be drawn together with a suture or two of fine catgut. The levator ani muscles should next be drawn together to their normal position in front of the rectum. This can be accomplished with a single suture of strong catgut. It is remarkable what a difference the one suture will make.

If the inverted V-incision has been employed the two branches of the wound are closed except in front, where a small area is left open for the gauze and tube drains.

### After-Treatment.

Irrigation is continued after the patient has returned to bed. A two-gallon porcelain tank with an outlet at the side is employed, and the flow is regulated by a clamp on the inlet tube. The outlet tube drains into a jar by the side of the bed. If the end is kept immersed in water air cannot get up the tube, and siphonage is obtained, thus keeping the bladder empty and preventing leakage around the perineal tubes.

A submammary infusion of $1\frac{1}{2}$ pints of salt solution is given either on the operating-table or after the return to bed. This is considered so valuable, both as a preventive to shock and anuria, and as a cure for post-operative thirst, that it is never omitted.

The gauze drains are removed on the day after the operation and no more packing put in. The tubes are pulled out a few hours later, and on the second day the patient is usually placed in a wheel-chair, and taken out of doors. No sounds are passed and stricture never results. Urotropin is administered early, and water is given in abundance. Within a few days the patient is generally walking about the hospital. Nothing is done to the wound except to keep it clean, and occasionally to cauterise exuberant granulations.

The subsequent convalescence has in the great majority of cases been remarkable, simple, and rapid. Out of 105 cases operated on during 1904 to 1906, only nine had persistence of the fistula for more than two months. Fifty per cent. of the cases did not remain in hospital longer than twenty-two days. In the great majority of cases urine passed through the penis during the first week, and inside of two weeks there was only a slight escape of urine through the perineal fistula.

## Figures and Tables

**Fig. 6. f1:**
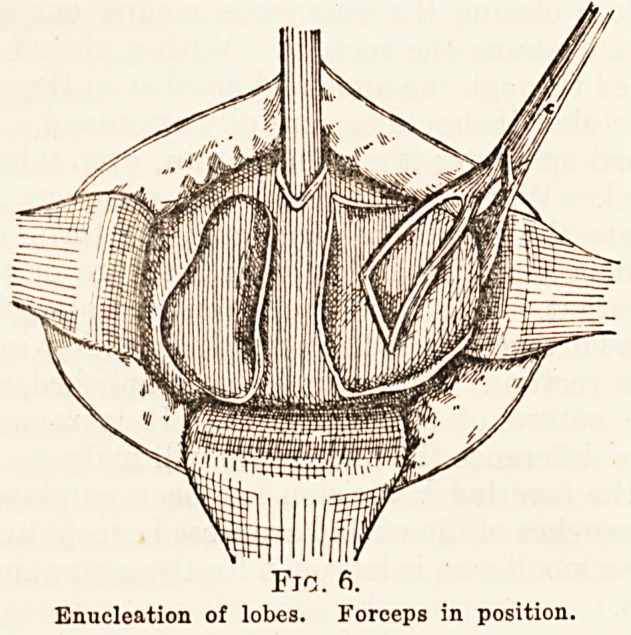


**Fig. 7. f2:**
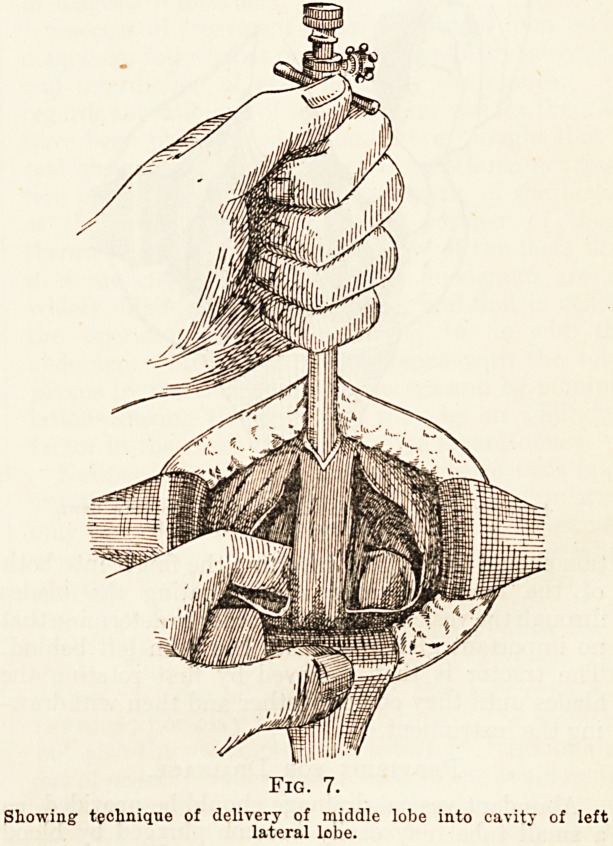


**Fig. 8. f3:**